# Relevant prognostic factors influencing outcome of patients after surgical resection of distal cholangiocarcinoma

**DOI:** 10.1186/s12893-018-0384-5

**Published:** 2018-08-13

**Authors:** Oliver Beetz, Michael Klein, Harald Schrem, Jill Gwiasda, Florian W. R. Vondran, Felix Oldhafer, Sebastian Cammann, Jürgen Klempnauer, Karl J. Oldhafer, Moritz Kleine

**Affiliations:** 1 Department of General, Visceral and Transplant Surgery, Medizinische Hochschule Hannover, Carl-Neuberg-Strasse 1, 30625 Hannover, Germany; 2 Core Facility Quality Management Transplantation, Integrated Research and Treatment Center Transplantation (IFB-Tx), Hannover Medical School, Hannover, Germany; 3 Department of General, Visceral and Oncological Surgery, Asklepios Klinik Barmbek, Hamburg, Germany

**Keywords:** Distal bile duct cancer, Extended surgery, Venous invasion, Preoperative biliary stenting

## Abstract

**Background:**

Distal cholangiocarcinoma (DCC) is a rare but over the last decade increasing malignancy and is associated with poor prognosis. According to the present knowledge curative surgery is the only chance for long term survival. This study was performed to evaluate prognostic factors for the outcome of patients undergoing curative surgery for distal cholangiocarcinoma.

**Methods:**

75 patients who underwent surgery between January 2000 and December 2014 for DCC in curative intention were analysed retrospectively. Potential prognostic factors for survival were investigated including the extent of surgery using purposeful selection of covariates in multivariable Cox regression modeling.

**Results:**

Preoperative biliary stenting (Hazard ratio (HR): 2.530; 95%-CI: 1.146–6.464, *p* = 0.020), the extent of surgery in case of positive histological venous invasion (HR: 1.209; 95%-CI: 1.017–1.410, *p* = 0.032), lymph node staging (HR: 2.183; 95%-CI: 1.250–3.841, *p* = 0.006), perineural invasion (HR: 2.118; 95%-CI: 1.147–4.054, *p* = 0.016) and postoperative complications graded in points according to Clavien-Dindo (HR: 1.395; 95%-CI: 1.148–1.699, *p* = 0.001) were indentified as independent significant risk factors for survival. Patients receiving preoperative biliary stenting showed prolonged duration between onset of symptoms and date of operation (*p* = 0.048).

**Conclusions:**

Preoperative biliary stenting reduces survival possibly due to delayed surgery. The extent of surgery is not an independent risk factor for survival except for patients with concomitant histological venous invasion. Oncological factors and postoperative surgical complications are independent prognostic factors for survival.

## Background

Cholangiocarcinoma is a malignant disease with poor prognosis and occurs with an incidence of 1.3 to 3.4 cases per 100,000 in Western countries [1].

The primary location of the carcinoma in reference to the biliary tract influences clinical manifestation and therapy. Therefore they are classified into intrahepatic, proximal extrahepatic and distal extrahepatic cholangiocarcinomas (DCC) [2].

DCC constitute up to 20–30% of all cholangiocarcinomas and are located between the cystic duct entry into the main hepatic duct and the Ampulla of Vater. Clinical symptoms comprise painless jaundice and/or weight loss [3].

Curative surgery is the only chance for long-term survival [3–9]. Palliative chemotherapy can achieve median survival rates of 8.1 to 11.7 months [10].

Apart from negative lymph node status, a tumor-free resection margin has been reported to have crucial impact on disease free survival [4, 5, 11, 12]. Nevertheless, tumor stage-specific surgical strategies have not been clearly defined. Some centers perform segmental bile duct resections when the tumor is small and located in the middle segment of the extrahepatic bile duct. Major surgery including partial pancreaticoduodenectomy is necessary to achieve tumor-free resection margins in the majority of cases [6, 13]. Some patients present with advanced tumors with additional infiltration of central bile ducts on one side of the liver or local infiltration of the portal vein. For these patients extended pancreatic resection with en-bloc portal vein resection or additional resection of proximal extrahepatic bile ducts might be necessary to achieve tumor-free resection margins [8, 14, 15].

Most published data describe inhomogeneous patient groups with respect to the primary location of biliary tract carcinoma [3–9]. The objective of this study was to evaluate the outcome of surgery in patients with DCC following resection in curative intent. In this context, the focus was put on the extent of surgical resection to clarify the role of organ preserving resection and extended surgery, including additional resection of the proximal extrahepatic bile ducts and/or partial resection of the portal vein.

## Methods

### Study cohort and investigated variables

This is a retrospective analysis of 75 patients with DCC undergoing surgical resection at the Department of General, Visceral and Transplant Surgery, Hannover Medical School, Germany between January 2000 and December 2014. Median overall survival follow-up was 19 months (range: 0–178 months). The clinical and histopathological variables of the study cohort are summarized in Table 1.

**Table 1:** Descriptive statistics of the investigated cohort of 75 patients undergoing surgery for distal cholangiocarcinoma

Variables			n (%)	Mean, Median (min.-max.)	Missing values n(%)
Age (years)			n.a.	65.2, 67 (33–87)	0 (0.0%)
Male gender			62 (82.7%)	n.a.	0 (0.0%)
Preoperative biliary stent			58 (77.3%)	n.a.	5 (6.7%)
Extent of surgery	Local bile duct excision w/o hilus	1 point	7 (9.3%)	3.0, 3 (1–5)	0 (0.0%)
	Local bile duct excision with hilus	2 points	3 (4.0%)		
	Pancreaticoduodenectomy	3 points	54 (72.0%)		
	with hilus resection	4 points	5 (6.7%)		
	with portal vein resection	5 points	6 (8.0%)		
Duration of operation (min)			n.a.	238.1, 230 (130–490)	0 (0.0%)
Grading	G1	1 point	2 (2.7%)	2.3, 2 (1–3)	0 (0.0%)
	G2	2 points	51 (68.0%)		
	G3	3 points	22 (29.3%)		
pT stages	pT1	1 point	4 (5.3%)	2.6, 3 (1–4)	0 (0.0%)
	pT2	2 points	28 (37.3%)		
	pT3	3 points	36 (48%)		
	pT4	4 points	7 (9.3%)		
pN stages	pN0	0 points	34 (45.3%)	0.6, 1 (0–2)	0 (0.0%)
	pN1	1 point	39 (52%)		
	pN2	2 point	2 (2.7%)		
M1 stage			5 (6.7%)	n.a.	0 (0.0%)
R0 resection			65 (86.7%)	n.a.	0 (0.0%)
Perineural invasion			46 (62.2%)	n.a.	1 (1.3%)
Venous invasion			16 (21.6%)	n.a.	1 (1.3%)
Lymph vessel invasion			22 (29.7%)	n.a.	1 (1.3%)
UICC stages	UICC Ia	1 point	4 (5.3%)	3.6, 4 (1–6)	0 (0.0%)
	UICC Ib	2 points	15 (20.0%)		
	UICC IIa	3 points	9 (12.0%)		
	UICC IIb	4 points	32 (42.7%)		
	UICC III	5 points	9 (12.0%)		
	UICC IV	6 points	6 (8.0%)		
Grading of complications	Clavien-Dindo Grade 0	0 points	25 (33.3%)	1.8, 1 (0–5)	2 (2.7%)
	Clavien-Dindo Grade I	1 point	15 (20.0%)		
	Clavien-Dindo Grade II	2 points	7 (9.3%)		
	Clavien-Dindo Grade III	3 points	11 (14.7%)		
	Clavien-Dindo Grade IV	4 points	9 (12.0%)		
	Clavien-Dindo Grade V	5 points	6 (8.0%)		
Complications leading to surgical intervention			19 (26.0%)	n.a.	2 (2.7%)
Observed overall survival in months			n.a.	29.1, 19 (0–145)	2 (2.7%)
Observed disease-free survival in months			n.a.	20.8, 13 (3–143)	47 (62.7%)
Hospital mortality			6 (8.0%)	n.a.	1 (1.3%)
Hospital stay in days			n.a.	29.8, 25 (7–102)	2 (2.7%)
ICU stay in days			n.a.	7.3, 3 (1–94)	17 (22.7%)
Days between onset of symptoms and date of surgery			n.a.	54.9, 34 (8–334)	36 (48.0%)

Table 1: Shown are descriptive statistics of the investigated cohort of 75 patients. The extent of surgery was scaled from 1 (local bile duct excision without resection of the perihilar bile ducts) to 5 points (pancreaticoduodenectomy including partial portal vein resection). This scale was used to calculate the variable “*extent of surgery graded in points multiplied by histological venous invasion*” as displayed in Table 2

### Inclusion criteria

Included were all resections for histologically confirmed DCC (*n* = 75) in patients older than 18 years of age. No exclusion criteria were defined.

### Definition of variables and surgical treatment

Due to a lack of histological differentiation between proximal and distal extrahepatic cholangiocarcinoma the diagnosis was defined by the location of the primary tumor distal to the confluence of the cystic duct and the common bile duct. According to intraoperative tumor extent patients either received local excisions of the extrahepatic bile ducts (including lymphadenectomy of the hepato-duodenal ligament), partial pancreaticoduodenectomy or pancreatic resection with additional partial resection of perihilar bile ducts, portal vein or liver. Oncological lymphadenectomy along the hepatic artery and down to the celiac trunk was performed in all patients receiving pancreatic resections. The extent of surgery was then graded in points as displayed in Table 1.

### Histopathology

DCC was histopathologically confirmed by paraffin embeddeded and haematoxylin and eosin (HE), Periodic acid–Schiff (PAS) and elastic Van Gieson’s (EVG) stained slides. In some cases additional immunohistochemical staining for IgG4 was performed. Tumors were classified according to the classification system proposed by the International Union Against Cancer [16].

### Study end-points

The primary study endpoints were overall survival (OS) and postoperative complications graded according to the Clavien-Dindo Classification of Surgical Complications [17]. Secondary endpoints were length of intensive care unit (ICU) and hospital stay.

### Statistical methods

The influence of nominal and ordinal variables on binary study endpoints were analysed with chi-squared test and Fisher’s exact test while the influence of continuous variables on these endpoints was analysed with univariable logistic regression. Median and mean values between groups were compared with the Mann-Whitney U test. Ordinal regression and Kaplan-Meier analyses with the Log-rank test were performed where appropriate.

Risk factors for patient survival were initially analysed with univariable Cox regression analysis.

Identification of independent risk factors influencing overall patient survival was achieved by developing a multivariable Cox regression model including potential multiplicative or additional factor interactions based on backwards likelihood elimination followed by forward likelihood inclusion of variables as has been proposed by Hosmer et al. and has been applied and published recently by our workgroup [18, 19].

The collected data was implemented and analysed using SPSS statistical software (version 23; SPSS Inc.; IBM corporation, Somers, NY) and JMP statistical software (version 13; SAS Institute; Cary, NC).

## Results

### Epidemiology and preoperative course

The median age of the study population at time of the operation was 67 years (33–87 years) and 82.7% (*n* = 62) of the patients were male (Table 1).

Symptoms were jaundice, epigastric pain, gastroesophageal reflux, weight loss, lack of appetite and nausea.

The standard preoperative assessment and staging included endoscopic retrograde cholangiopancreatography (ERCP) received by 88.0% of the patients, ultrasound (78.7%) and computed tomography (CT) (74.7%) or magnetic resonance imaging (17.3%). Location of the tumor was primarily defined by ERCP. The extent of tumor was anticipated mainly according to CT, however CT scans could not identify any tumor mass in 22 patients. Five of these patients underwent additional MR tomography for verification of tumor extent with successful detection of the lesions in two patients.

Biopsies were performed in 33 patients (44.0%) whereas brush cytologies were performed in 39 patients (52.0%). Hereby the diagnosis was verified in 49.3% of the patients.

**Fig. 1:**
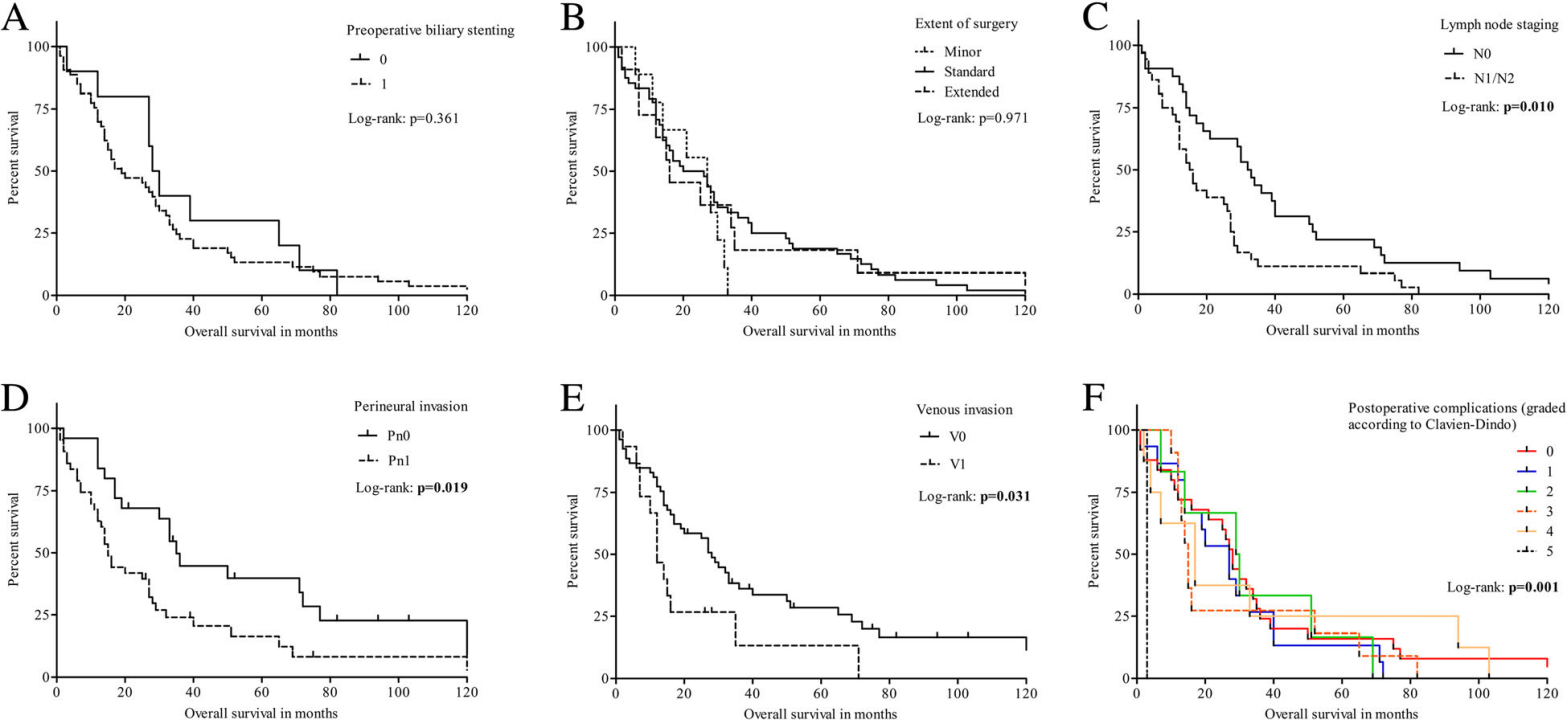
Survival (Kaplan–Meier) after resection of distal cholangiocarcinoma according to preoperative biliary stenting (**a**), extent of surgery (**b**), lymph node staging (**c**), perineural invasion (**d**), venous invasion (**e**) and postoperative complications graded by Clavien-Dindo (**f**). Shown are all risk factors that were significant in univariable or multivariable Cox regression analysis, respectively. Overall survival in months after resection of distal cholangiocarcinoma depending to the studied factors

Preoperative biliary stenting via ERCP (internal drainage) as a consequence of relevant cholestasis was performed in 77.3% of the patients (*n* = 58). Percutaneous Transhepatic Cholangio-Drainage (PTCD) was not required in our patient collective. Patients receiving biliary stenting prior to operation showed significantly prolonged duration between onset of symptoms and operation (*p* = 0.048). Preoperative biliary stenting was found to be an independent risk factor for worse overall survival in multivariable Cox regression analysis (HR: 2.530; 95%-CI: 1.146–6.464, *p* = 0.020) (Table 2) (Fig. 1a).

**Table 2:** Shown are the uni- and multivariable Cox regression analysis to identify risk factors for overall survival

Variables (univariable Cox regression analysis)			HR	95%-CI	p-value
Age (years)			1.005	0.980 – 1.032	0.713
Male gender			0.791	0.431 – 1.569	0.482
Preoperative biliary stent			1.412	0.704 – 3.238	0.350
Extent of surgery	Local bile duct excision w/o hilus	1 point	1.034	0.753 – 1.427	0.836
	Local bile duct excision with hilus	2 points			
	Pancreaticoduodenectomy	3 points			
	with hilus resection	4 points			
	with portal vein resection	5 points			
Perihilar bile duct resection			0.439	0.132 – 1.081	0.077
Partial portal vein resection			2.070	0.786 – 4.534	0.129
Duration of operation (min)			0.998	0.994 – 1.001	0.311
Grading	G1	1 point	1.638	0.921 – 2.863	0.092
	G2	2 points			
	G3	3 points			
pT stages	pT1	1 point	1.191	0.855 – 1.656	0.300
	pT2	2 points			
	pT3	3 points			
	pT4	4 points			
pN stages	pN0	0 points	1.717	1.092 – 2.668	**0.020**
	pN1	1 point			
	pN2	2 point			
M1 stage			0.861	0.259 – 2.119	0.769
R0 resection			0.733	0.376 – 1.602	0.411
Perineural invasion			1.913	1.110 – 3.397	**0.019**
Venous invasion			1.932	1.006 – 3.502	**0.048**
Lymph vessel invasion			1.635	0.904 – 2.850	0.102
UICC stages	UICC Ia	1 point	1.119	0.931 – 1.346	0.230
	UICC Ib	2 points			
	UICC IIa	3 points			
	UICC IIb	4 points			
	UICC III	5 points			
	UICC IV	6 points			
Grading of complications	Clavien-Dindo Grade 0	0 points	1.226	1.034 – 1.452	**0.020**
	Clavien-Dindo Grade I	1 point			
	Clavien-Dindo Grade II	2 points			
	Clavien-Dindo Grade III	3 points			
	Clavien-Dindo Grade IV	4 points			
	Clavien-Dindo Grade V	5 points			
Complications leading to surgical intervention			1.302	0.696 – 2.306	0.395
Variables (final multivariable Cox regression model)					
Preoperative biliary stenting			2.530	1.146 – 6.464	**0.020**
Venous invasion (yes=1, no=0) multiplied by Extent of surgery graded in points			1.209	1.017 – 1.410	**0.032**
Lymph node staging graded in points			2.183	1.250 – 3.841	**0.006**
Perineural invasion			2.118	1.147 – 4.054	**0.016**
Complications graded by Clavien-Dindo			1.395	1.148 – 1.699	**0.001**

Univariable Cox regression analysis identified lymph node staging (N), perineural invasion, venous invasion and grading of complications according to Clavien-Dindo as significant risk factors for worse overall survival

The final multivariable Cox regression model determined preoperative biliary stenting, the extent of surgery in case of positive histological venous invasion, lymph node staging and perineural invasion as well as postoperative complications graded in points according to Clavien-Dindo as independent significant risk factors for survival

The median time interval between onset of symptoms and operation was 34 days (Table 1).

### Surgical results

In ten patients local excisions of the extrahepatic bile ducts were performed which included lymphadenectomy of the hepato-duodenal ligament representing a collective undergoing rather minor extent of surgery.

26 patients underwent pylorus-preserving partial pancreaticoduodenectomy and 28 patients underwent classical partial pancreaticoduodenectomy without preservation of the pylorus.

Six patients underwent additional partial portal vein resection and three patients underwent additional resection of proximal/central extrahepatic bile ducts a in addition to pancreaticoduodenectomy. One patient underwent right trisectorectomy of the liver (Segments 1, 4–8) and perihilar bile duct resection in addition to classical partial pancreaticoduodenectomy.

To analyse the influence of the extent of the surgical approach on patient outcome we graded the aforementioned procedures according to Table 1. Univariable analysis did not show significant influence on survival (Table 2, Fig. 1b). Multivariable analysis however displayed significant influence on survival in case of concomitant venous invasion (HR: 1.209; 95%-CI: 1.017–1.410, *p* = 0.032) (Table 2).

Since resection of the perihilar bile duct bifurcation, due to proximal tumor infiltration of the resection margin, was performed in seven patients we separately analysed postoperative outcome of these cases. Tumor-free resection margins were diagnosed in final histology in four of these seven patients and none developed anastomotic leakage. Median overall survival (28.0 months) was longer as compared to all other patients (16.5), however Cox regression analysis revealed no significant influence on survival (*p* = 0.077).

Segmental portal vein resection due to tumor infiltration, representing a rather extended approach of surgical resection, was performed in six patients in total (8%). Vein reconstruction was achieved by primary suture in five and by insertion of a ringed polytetrafluoroethylene (PTFE) graft in one case. Portal vein thrombosis occurred in one patient and was treated by thrombectomy. Following additional portal vein resection all patients died during follow-up with a lower median overall survival (13.5 versus 21.0 months). Accordingly, Cox regression analysis revealed a negative impact of portal vein infiltration, although not statistically significant (*p* = 0.129).

Negative resection margins (R0) in final histology were achieved in 86.7% of the patients. Residual tumor did not significantly influence overall survival in uni- or multivariable analysis as displayed in Table 2.

Initial intraoperative resection margins revealed residual tumor in 24 patients (32.0%). In eleven of these patients tumor free resection margins could be achieved by further local resection of adjacent tissue and in three patients by an extension of the surgical approach, i.e. pancreatic and hepatic resection respectively.

In the remaining patients further resection was not possible due to infiltration of superior mesenteric artery and/or due to the patients reduced general health condition respectively.

Seven of the ten patients undergoing local excision of the bile duct showed residual tumor in the initial intraoperative resection margins. Residual tumor was localized at the distal resection margin in six patients with simultaneous infiltration of the proximal resection margin in one patient. In one patient residual tumor was detected at the proximal resection margin. Further local resection ultimately led to tumor free resection margins in all but three patients.

Patients with tumor free resection margins in the initial intraoperative frozen sections did not show significantly better outcome regarding recurrence or survival when compared to patients undergoing further local or extended resection in order to achieve tumor free resection margins in final histopathological analysis (*p* = 0.092, *p* = 0.903 respectively).

### Histopathological results

Low T-staging depended significantly on a short time period between first symptoms and operation (*p* = 0.021, ordinal regression). However, delayed surgery did not significantly influence tumor stage according to UICC (*p* = 0.160, ordinal regression) or the success of the operation defined as tumor-free resection margins (Odds ratio (OR): 0.995; 0.974–1.017; *p* = 0.643, logistic regression).

Lymph node infiltration (N1/N2), detected in 41 patients (54.7%), was a significant risk factor for worse overall survival in univariable (*p* = 0.020) and multivariable analysis (HR: 2.183; 95%-CI: 1.250–3.841, *p* = 0.006) (Fig. 1c).

In 46 patients (62.2%) histopathological analysis revealed perineural invasion. Univariable (*p* = 0.019) and multivariable analysis (HR: 2.118; 95%-CI: 1.147–4.054, *p* = 0.016) identified perineural invasion as significant risk factor for overall survival (Fig. 1d).

Venous invasion was observed in 16 patients (21.6%) with significant influence on worse overall survival in univariable analysis (*p* = 0.048) (Fig. 1e). Further histopathological results and their influence on patient outcome are displayed in Tables 1 and 2.

### Postoperative course and complications

Median postoperative hospital stay was 25 days including a median of 3 days in the ICU. Extended surgery did not significantly prolong postoperative hospital stay nor time spent in the ICU.

Fourty-five patients (60.0%) had complications with varying degrees of severity graded according to Clavien-Dindo (Tables 1 and 3). Univariable (*p* = 0.020) and multivariable analysis (HR: 1.395; 95%-CI: 1.148–1.699, *p* = 0.001) identified the grade of postoperative complications as significant risk factor for worse overall survival (Fig. 1f).

**Table 3:** Complications in patients with distal cholangiocarcinoma after receiving surgical treatment

Complications	number of patients (%)
Wound infection	16 (21.3)
Pancreatic fistula grade B	10 (13.3)
Insufficiency of biliodigestive anastomosis	8 (10.7)
Pancreatic fistula grade C	7 (9.3)
Death within 30 postoperative days	5 (6.7)
Renal insufficiency	5 (6.7)
Delayed gastric emptying	5 (6.7)
Erosion haemorrhage	4 (5.3)
Acute confusional state	3 (4.0)
Pleural effusion	3 (4.0)
Pneumonia	2 (2.7)
Cardiovascular events	2 (2.7)
Urinary tract infection	1 (1.3)
Hepatic insufficiency	1 (1.3)
Thrombosis of portal vein	1 (1.3)
Intrabdominal abscess	1 (1.3)
Postoperativ bleeding	1 (1.3)

Table 3: Number (and frequency) of complications in patients with distal cholangiocarcinoma after receiving surgical treatment

Patients undergoing different extents of surgery as stated above did not experience statistically significant differences regarding rate and severity of postoperative complications.

The most common complications were wound infections with a rate of 21.3%.

Type A pancreatic fistulas were not documented due to missing data on enzyme measurements from abdominal drains. Type B pancreatic fistulas were observed in ten patients (13.3%). Type C pancreatic fistulas were diagnosed in seven patients (9.3%) and total pancreatectomy was performed in six individuals (8.0%).

Eight patients (10.7%) suffered from insufficiency of the biliodigestive anastomosis.

Early mortality, defined as death in the first 30 post-operative days was 6.7% (*n* = 5). Causes of death were cardiac arrest due to cardiac infarction in one patient, sepsis and organ failure after total pancreatectomy due to insufficiency of the pancreatic anastomosis and arterial bleeding in two patients, and organ failure due to severe pneumonia in two further patients.

After local excision of the extrahepatic bile duct three of ten patients suffered from local tumor recurrence while another patient developed liver metastasis in the observation period. Patients undergoing standard pancreatic resection developed tumor recurrence in 21 documented cases ranging from local recurrence (*n* = 5), to lymph node, liver, bone and peritoneal metastases (*n* = 16). Three of eleven patients after extend pancreatic surgery suffered from local recurrence whereas two patients developed distant metastases in liver and peritoneum.

The rate of observed tumor recurrence was not significantly influenced by the extent of surgery.

The median overall survival was 19 months (0–145). Fifty-two (72.0%), 18 (30.0%) and 11 (22.0%) patients survived more than 1, 3 and 5 years, respectively. Thirteen patients (17.3%) were still alive at the time of data analysis.

## Discussion

Due to the aforementioned comparatively low incidence of DCC among the general population, especially from Western countries, reports on outcome after surgery, of large patient collectives, are scarce. This data from a single high-volume center was analysed to identify prognostic factors for unfavourable patient outcome.

Interestingly no published data investigated preoperative stenting in patients with DCC and its impact on survival yet. In multivariable analysis we identified preoperative stenting as strongest risk factor for survival. A significantly longer duration between first symptoms and surgery in these patients could provide a clinically plausible explanation for the inferior outcome (Table 2). A prospective trial aiming to elucidate the role of preoperative biliary stenting in patients with pancreatic head cancer showed a significantly higher rate of complications after preoperative stenting [20]. So far similar observations have not yet been reported for DCC. The current study did not find a significant influence of biliary stenting on the rate or severity of postoperative complications. Nonetheless, we are well aware that preoperative conditions, which were not analysed in the current study, like cholangitis or severe biliary obstruction which require prolonged biliary drainage could be an explanation for the inferior postoperative outcome [21].

Survival after surgical treatment in the current study was similar to several previous studies [22–28].

Reported outcomes of patients with DCC after extended pancreatic surgery are extremely scarce. Solely Courtin-Tanguy et al. investigated extended surgery for treatment of DCC and found that combined organ resection was an independent risk factor for worse survival [29]. Interestingly, the present study showed that extended pancreatic resection was not significantly associated with inferior survival except in case of concomitant histological venous invasion as displayed by the identified interaction variable. Previous studies on risk factors for survival have not taken possible multiplicative factor interactions into account [28, 30]. The approach to multivariable Cox regression in this study deploys the previously published methodology for the detection of such factor interactions [18]. However, the displayed factor interaction is not suitable for intraoperative decision making regarding the extent of surgery due to the circumstance that histopathogical results verifying microscopic venous invasion are rarely available pre- or intraoperatively. Instead the prognostic value of venous invasion in patients undergoing extended surgery should be considered within the postoperative therapeutic strategy.

Partial portal vein resection due to macroscopic tumor invasion had no statistically significant influence on survival in Cox regression analysis (Table 2) in this study while several studies described poor survival for patients after portal vein resection, although only Miura et al. were able to show significant influence on survival in multivariable analysis [28, 31, 32]. This discrepancy could be due to the small number of cases in this study since a tendency towards worse survival in patients after partial portal vein resection has been observed. The benefit of portal vein resection therefore remains unclear.

Intraoperative frozen sections were crucial for flexible intraoperative decision making on the extent of resection, achieving tumor-free resection margins and partially led to an extension of the surgical strategy including partial portal vein, additional liver or perhilar bile duct resection. This is emphasized by the fact that the preoperative CT scans in the displayed cohort were not a reliant predictor of tumor extent. CT scans of 22 patients identified no measurable tumor mass although final histology revealed tumor sizes between T stage 1 and 4 in these patients. Even additional MR tomography did not provide significantly better results in preoperative detection of tumor extent. Therefore, in some cases endosonography might help for identification of tumor extent.

Finally patients with stricture of the distal bile duct suspicious of a malignant tumor should be explored surgically even if histology was negative, since 49.3% of preoperative histologies were false negative.

The impact of positive tumor infiltration at the surgical resection margin (R1/2 resection) on postoperative survival following curative resection of DCCs is widely discussed. We found no significant impact of R1/2 resection on overall survival in Cox regression analysis (Table 2). This finding is in line with some of the published series [15, 33–35]. Other studies identified positive resection margins as an independent prognostic factor on overall survival [3, 28]. Interestingly the percentage of R1 or R2 resected patients differs widely between the aforementionend published series from 7.0 to 43.3% [3, 15, 28, 33–37]. Our series shows a comparatively low rate of R1/R2 resections with 13.3%. A comparable retrospective study with 10.5% positive resections margins found no significant difference in survival for these patients in uni- and multivariable analysis [15]. Two published series found rates of 13.4 and 14.0% of R1/2 resected patients, respectively with significant influence on patient survival in uni- but not in multivariable analysis [33, 35]. In contrast series with higher rates of cancer positive resection margins identified R1/2 resections as an independent significant negative prognostic factor on survival in multivariable analysis [28, 38]. This could implicate a correlation of higher rates of positive resection margins with the identification of R1/2 resections as a significant negative prognostic factor in multivariable analysis in some series, which in turn would explain the conflicting results of our and these series [15, 33–35]. As displayed above, further local or extended resection in case of initially positive intraoperative resection margins was performed on a regular basis especially in patients undergoing local excision of bile duct and did not impact recurrence rate or overall survival. Interestingly there is presently no further data on this matter in patients undergoing resection for distal cholangiocarcinoma. Until further clarification the primary goal of surgical resection of DCC should be to achieve R0 resection. We therefore recommend intraoperative frozen sections of the resection margins and when residual disease is detected, additional resection of the bile duct bifurcation or locally infiltrated structures should be performed if technically feasible.

The presence of tumor positive lymph nodes was identified as a significant prognostic factor in multivariable analysis (Table 2) as was reported by different meta-analysis and some multicenter studies [28, 30, 36, 38]. Apart from a German study by Petrova et al., all mentioned publications reported a significant impact of lymph node metastasis on survival in uni- and not in multivariable analysis [28]. The above mentioned German colleagues did not report on the impact of the number of metastatic lymph nodes on survival. This seems to be relevant as Kiriyama et al. demonstrated earlier that the total number of infiltrated lymph nodes influences patients outcome. They found a significant difference when comparing no lymph node metastasis with 1–3 and more than 4 metastases [39]. This correlates well with our findings, as we classified the number of lymph node metastasis according to the newest TNM 8th edition classification for DCC in N0 versus N1 (1–3 positive lymph nodes) versus N2 (≥ 4 positive lymph nodes) resulting in a negative prognostic factor for each grade with a hazard ratio of 2.18 in multivariable analysis.

Postoperative complications graded in points according to Clavien-Dindo had a significant impact on patient outcome (Table 2). The influence of postoperative morbidity on the outcome of patients with DCC was recently observed by Petrova et al. and Andrianello et al.. Aside from direct consequences leading to early death in hospital it is assumed that patients with complicated postoperative course experience delay in the start of adjuvant therapy options (i.e. chemotherapy) resulting in an inferior survival [28, 40]. However the efficiency of adjuvant chemotherapy in patients with cholangiocarcinoma, as currently investigated by the multicenter prospective ACTICCA trial, remains unclear [41].

## Conclusion

Preoperative biliary stenting, positive lymph node staging, perineural invasion as well as extended surgery in patients with concomitant venous invasion and postoperative complications should be considered within the perioperative therapeutic strategy. It is important to state that it is difficult to draw definite conclusions from this retrospective study considering the small patient collective undergoing extended surgery, respectively. Therefore it would certainly be beneficial performing multi-center studies with prospective analysis to determine which surgical approach is justifiable regarding optimal patient outcome. In addition the effects of preoperative biliary drainage in patients with DCC and relevant cholestasis on survival should be thoroughly examined in respect of our presented data.

## Abbreviations

DCC: Cholangiocarcinoma
EVG: Elastic Van Gieson’s
HE: Haematoxylin and eosin
HR: Hazard ratio
ICU: Intensive care unit
OR: Odds ratio
OS: Overall survival
PAS: Periodic acid–Schiff
PTFE: Polytetrafluoroethylene
